# The symbioses of endophytic fungi shaped the metabolic profiles in grape leaves of different varieties

**DOI:** 10.1371/journal.pone.0238734

**Published:** 2020-09-11

**Authors:** Xiao-Xia Pan, Ming-Quan Yuan, Si-Yu Xiang, Yin-Min Ma, Ming Zhou, You-Yong Zhu, Ming-Zhi Yang

**Affiliations:** 1 School of Agriculture, Yunnan University, Kunming, China; 2 School of Chemistry and Environment, Yunnan MinZu University, Kunming, China; 3 School of Chemistry and Chemical Engineering, Yunnan University, Kunming, China; 4 School of Life Science, Yunnan University, Kunming, China; 5 School of Agronomy, Yunnan Agricultural University, Kunming, China; 6 School of Ecology and Environmental Science, Yunnan University, Kunming, China; Fujian Agriculture and Forestry University, CHINA

## Abstract

Endophytic fungi produce many novel bioactive metabolites that are directly used as drugs or that function as the precursor structures of other chemicals. The metabolic shaping of endophytes on grape cells was reported previously. However, there are no reports on the interactions and metabolic impact of endophyte symbiosis on *in vitro* vine leaves, which may be examined under well-controlled conditions that are more representative of the natural situation of endophytes within grapevines. The present study used an *in vitro* leaf method to establish endophyte symbiosis of grapevines and analyze the effects on the metabolic profiles of grape leaves from two different cultivars, ‘Rose honey’ (RH) and ‘Cabernet sauvignon’ (CS). The effects of endophytic fungi on the metabolic profiles of grape leaves exhibited host selectivity and fungal strain specificity. Most of the endophytic fungal strains introduced novel metabolites into the two varieties of grape leaves according to the contents of the detected metabolites and composition of metabolites. Strains RH49 and MDR36, with high or moderate symbiosis rates, triggered an increased response in terms of the detected metabolites, and the strains MDR1 and MDR33 suppressed the detected metabolites in CS and RH leaves despite having strong or moderate symbiosis ability. However, the strain RH12 significantly induced the production of novel metabolites in RH leaves due to its high symbiosis ability and suppression of metabolites in CS leaves.

## Introduction

The term “endophyte” was introduced for the first time by de Bary in 1866 [1], and it is accepted virtually worldwide as indicating “microorganisms that live within plant tissues at some time in their life cycle without causing apparent symptoms of disease in their hosts” [2]. Recent researchers tended to define the term “endophytes” as indicating all microorganisms that colonize plant tissues internally for all or part of their lifetime and refers only to the habitat of the organisms and not their function [3]. Various factors affect grapevine microbial communities, including anthropogenic factors [4], plant physiology [5], the environment [6, 7], and pathogen infections [8–10]. Endophytes are composed of fungi, bacteria and actinomycetes [11]. Fungal endophytes are found ubiquitously in all studied plants [12, 13]. Fungal endophytes can synthesize various bioactive metabolites and have become research hot spots in the fields of microbiology, botany, pharmacy and agronomy [14–17].

Grapevines are the most cultivated fruit plant worldwide and are one of the most important crops from an economic standpoint. Grapevines are also considered a source of health-promoting secondary metabolites [18]. Grapevines harbor diverse microorganisms that are the source of the ‘terroir’ of grape wine qualities and characteristics [19]. During the life of grapevines, endophytic fungi play important roles as beneficial microorganisms or pathogens. Many studies on the fungal grapevine community were reported, but exactly how the fungal community coexists within the plant and influences the ‘terroir’ of grapes is not known. Due to the complex endophytic communities within grapevines, it is difficult to determine which fungi determine grape metabolism *in vivo*. Therefore, the present study used an *in vitro* leaf method to establish endophyte symbiosis of grapevines. Fourteen strains of endophytic fungi isolated from vine leaves of ‘Rose honey’ (RH) were co-cultured with grape leaves of ‘Cabernet sauvignon’ (CS) and RH and the changes in general and in specific metabolites in grape leaves were analyzed using high-pressure liquid chromatography (HPLC).

## Materials and methods

### Preparation of aseptic grapevine leaves

Tissue-cultured aseptic grapevine seedlings (single bud clones, *Vitis vinifera* L. cultivar: *Vitis vinifera* cv. CS and *V*. *Vinifera* L. × *V*. *labrusca* L. RH) were cultured for 40–50 days with 6 to 7 expanded leaves. Aseptic leaves from grapevine seedlings were harvested at the optimal stage for susceptibility for bioassays. Four to five immature leaves were collected from shoots with petioles (approximately 0.5 cm long) and rinsed with sterile distilled water.

### Preparation of endophytic fungal strains

Endophytic fungal strains (Table 1) were isolated from the leaves of grape cultivars of RH in local vineyards (Yunnan Province, China) and used in all experiments in this study. The isolation of fungal endophytes followed the tissue patch method [20], and purified fungal strains were identified using ITS DNA sequences [21]. The fungal strains used to establish endophyte-host symbionts were plate-cultured on glass paper that covered a potato dextrose agar medium in Petri dishes for one week, and fungal mycelia were fully suspended in 0.9% normal saline (final concentration was 10 g/L).

**Table 1:** Endophytic fungal strains used in the experiment.

Strain ID	Species	Strain ID	Species
RH7	*Epicoccum nigrum*	RH48	*Colletotrichum gloesporioides*
RH12	*Nigrospora oryzae*	RH49	*Fusarium fujikuroi*
RH32	*Alternaria*	MDR1	*Nigrospora oryzae*
RH34	*Trichothecium roseum*	MDR3	*Fusarium oxysporum*
RH36	*Fusarium verticillioides*	MDR4	*Fusarium annulatum*
RH44	*Alternaria arborescens*	MDR33	*Colletotrichum gloeosporioides*
RH47	*Fusarium proliferatum*	MDR36	*Colletotrichum siamense*

### Establishing the symbionts of fungal endophytes and *in vitro* grapevine leaves

To establish endophytes-grape leaves symbionts *in vitro*, detached leaves were smear inoculated with a fungal mycelium suspension. Leaves were placed on the surface of 0.9% water agar plates with the petiole inserted into the water agar. The Petri dishes were immediately sealed with parafilm, and the plates were incubated at 25°C under 12-h photoperiods for 15 days. Two or three leaves were assayed per treatment, and the experiments were repeated three times.

### Isolation of the fungal endophytes

To determine the symbiosis rates of fungal endophytes of *in vitro-*infected grape leaves, leaves with different treatments were harvested 15 days after inoculation, and each leaf was cut into two parts along the main vein. One leaf part was for HPLC assay, and the other part was used to detect the symbiosis rates of fungal endophytes. Surface sterilization of leaves was performed using 75% ethanol for 30 s, 3% sodium hypochlorite for 20 min, and three washes with sterilized water. After surface sterilization, the leaves were cut using sterile scissors into 0.5-cm segments and placed on the isolation medium (potato dextrose agar medium). Symbiosis rates were calculated as the percentage of emerged fungal colonies per leaf patch and used to describe the efficiencies of symbiosis of fungal endophytes. Fungal colonies were identified using ITS DNA sequences.

### HPLC assay

For HPLC assays, the leaves and fungal endophytes were dried at 110°C for 10 min, then at 60°C for 2 or 3 days to a constant weight. The leaves and fungal endophytes were ground into a powder, and 100 mg of the dried powder was accurately weighed, extracted with 1 mL of methanol (contains 0.1% of hydrochloric acid) for 12 h, and sonicated for 60 min. The extracts were centrifuged for 10 min at 4,000 rpm at 4°C, and supernatants were filtered with 0.45-μm filter columns. The filtrates (10 μL) were uploaded for analyses on a reversed-phase C18 column (Thermo) using an HPLC instrument (Agilent, USA). The elution phase was acetonitrile (Sigma, St. Louis, MO, USA): methanol: water (A: B: C) = 95:0.5:4.5 and detected with a UV detector at 254 nm. The elution speed was 1 mL/min, and the column temperature was 30°C. Samples were eluted using the gradient procedures illustrated in S1 Table. The content of metabolites (mg/g) was quantified using catechin as a standard with an R^2^ = 0.999.

### Statistical analysis

Data were analyzed using Microsoft Excel. Values are presented as the means of three replicates (means ± SD) for each treatment. One-way analysis of variance (ANOVA) and Tukey’s HSD tests were performed using SPSS software to determine the significance of the differences between the samples at the *P* ≤ 0.05 level. Treatments were subjected to Squared Euclidean distance Hierarchical clustering using SPSS 16.0 software. Heat-maps were generated in Microsoft Excel 2013 according to the content of the detected metabolites. Principal component analysis (PCA) was analyzed in the R package (Version 3.6.1).

## Results

**Fig 1:**
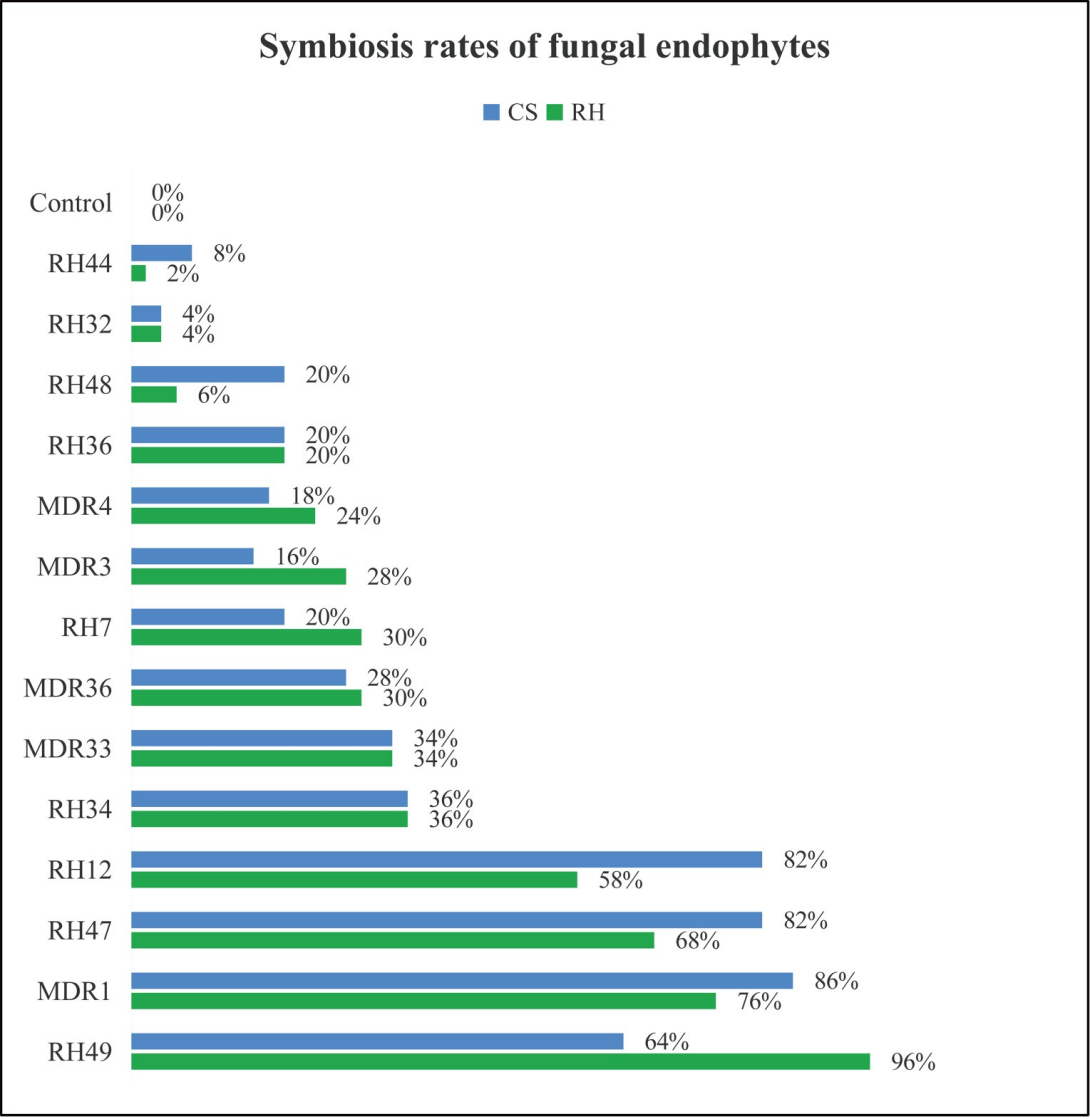
Symbiosis rates of fungal endophytes of *in vitro* grape leaves after infection with endophytic fungal strains.

Detached leaves were smear inoculated with fungal mycelium suspension (final concentration was 10 g/L) for establishing endophytes-grape leaves symbionts *in vitro*. Smear inoculation could successfully lead to the infection and symbiosis of fungal endophytes in aseptic grape leaves *in vitro* and most of the leaves underwent fungi infections maintained good physiological conditions (Fig 1). Symbiosis rates of fungal endophytes in *in vitro* grape leaves of both grapevine cultivars ranged from 4% to 96% (Fig 1), which were calculated as the percentage of emerged fungal colonies per leaf patch and used to describe the efficiencies of symbiosis of fungal endophytesFungal strains RH12 (*Nigrospora* sp.), RH47 (*Fusarium* sp.), RH49 (*Alternaria* sp.) and MDR1 (*Nigrospora* sp.) demonstrated strong ability of symbiosis to RH and CS grape leaves. Fungal strains RH32 (*Alternaria* sp.), RH44 (*Alternaria* sp.) and RH48 (*Colletotrichum* sp.) exhibited weak symbiosis entry into the leaves of the two grapevine cultivars (Fig 1).

**Fig 2:**
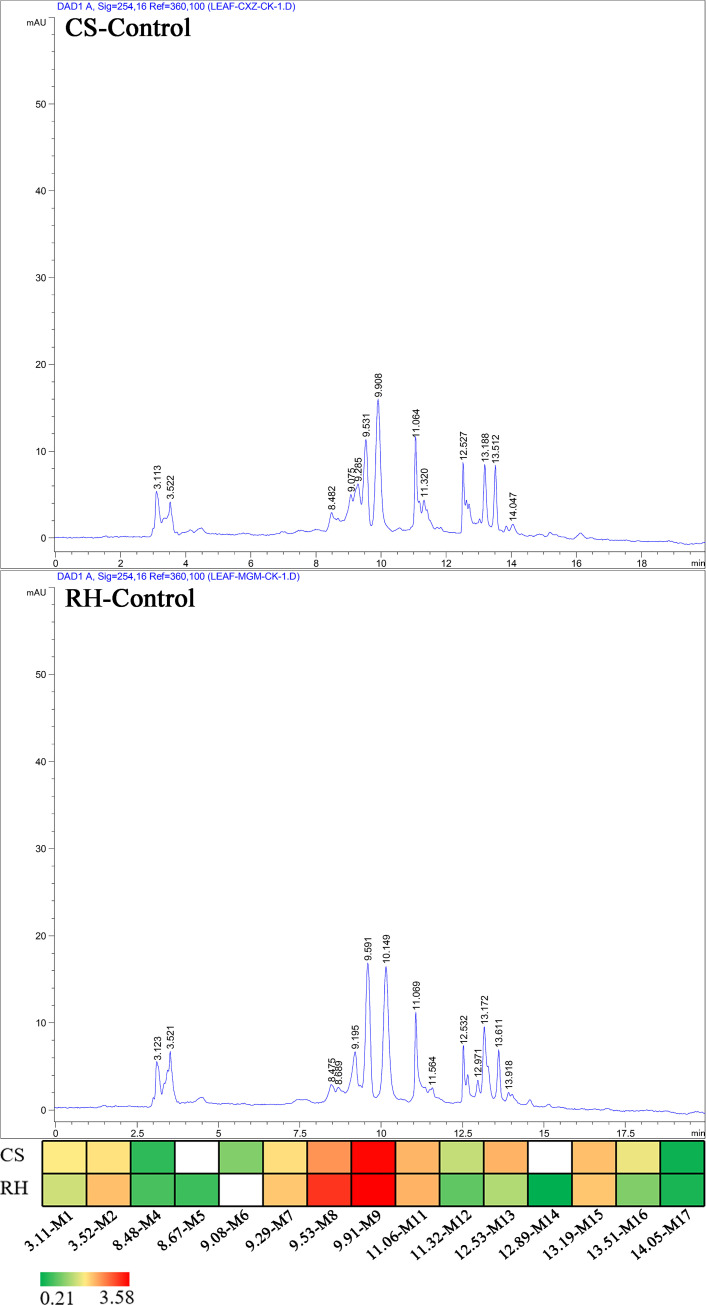
The basic metabolite profiles of CS and RH grape leaves using HPLC assays.

Similar metabolite profiles were detected in grape leaves from CS and RH cultivars in HPLC assays (Fig 2). Thirteen and fourteen metabolites in CS and RH grape leaf methanol extracts were isolated, respectively. The concentrations of the detected metabolites varied from 0.26 mg/g to 3.53 mg/g in CS grape leaves and 0.21 mg/g to 3.58 mg/g in RH leaves. Twelve metabolites were detected in CS and RH leaves. Only metabolite M6 was specifically detected in CS leaves, and two specific metabolites, M5 and M14, were only detected in RH leaves.

**Fig 3:**
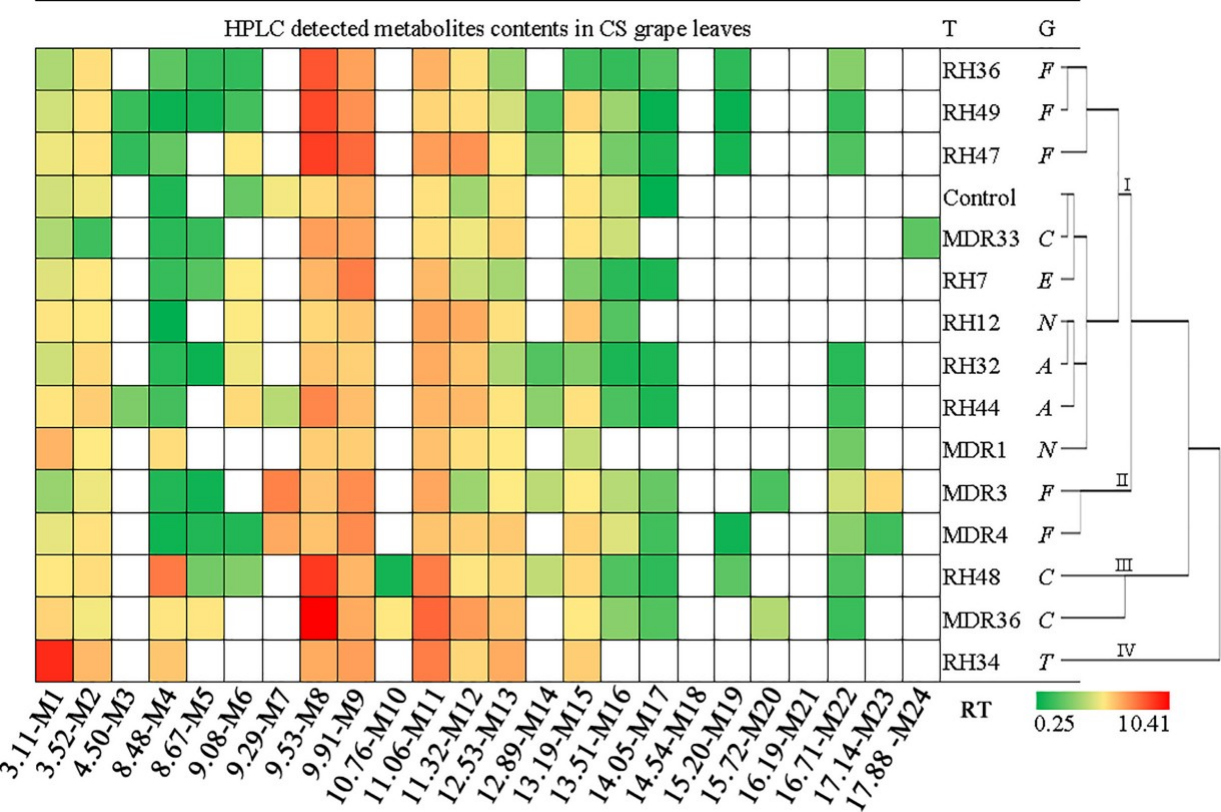
Heatmap and clustering of HPLC-detected metabolite contents in CS grape leaves. T: treatment (represented as endophytic fungal strain ID and the control). HPLC-detected compounds are marked as colored bricks, and different colors represent the content of the metabolites. G: genus of the endophytic fungal strains, *C*: *Colletotrichum; E*: *Epicoccum; A*: *Alternaria; F*: *Fusarium; T*: *Trichothecium; N*: *Nigrospora*. RT: the retention time that the metabolites appeared in HPLC assay.

**Fig 4:**
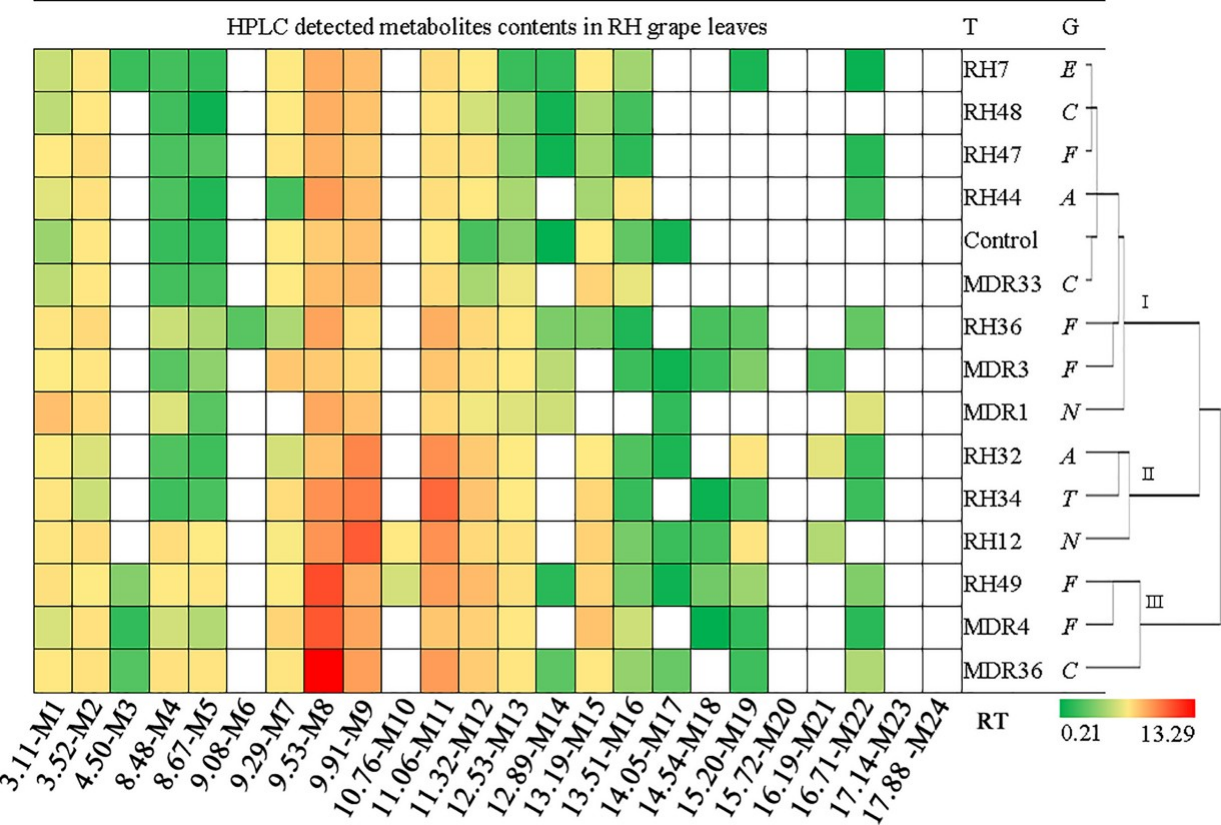
Heatmap and clustering of HPLC-detected metabolite contents in RH grape leaves.

Notwithstanding the similarity of the basic metabolite profiles of CS and RH leaves, the composition of the detected metabolites in grape leaves was differentially modified due to the symbiosis of endophytic fungi. The detected metabolites in CS leaves covered retention times from 3.11 min to 17.88 min, whereas metabolites in RH leaves appeared from 3.11 min to 16.71 min (Figs 3 and 4, S1 and S2 Figs). Treatments with endophytic fungal strains caused the numbers of detected metabolites to vary from 9 to 17 in CS leaves and 12 to 17 in RH leaves (Figs 3 and 4, Table 2). The concentration of individual metabolites in CS grape leaves varied from 0.25 mg/g to 10.41 mg/g, and the detected metabolite contents in RH leaves varied from 0.21 mg/g to 13.29 mg/g (S2 and S3 Table). The chromatograms revealed that the symbiosis of endophytic fungi exclusively reshaped the metabolic profiles in grape leaves compared to leaves with no endophyte symbiotic leaves (Figs 3 and 4, Table 2). Clustering of the biological replicates of all treatments to CS and RH leaves based on the appearance and absence of detected metabolites, replicates of one treatment tended to cluster together (S3 and S4 Figs).

**Table 2:** The impacts of endophytic fungal infectionon special parameters of metabolites.

Leaves	Treatment	Numbers of total metabolites detected	Numbers of novel metabolites detected	Numbers of suppressed metabolites	Total contents of detected metabolites (mg/g FW)	Retention time of maximum metabolites (min)	Contents of maximum metabolites (mg/gFW)
CS	Control	13			17.45	9.91	3.53
	RH7	13	1	1	20.73	9.91	5.55
	RH12	11	0	2	22.33	11.06	4.01
	RH32	15	3	1	20.39	11.06	3.81
	RH34	9	0	4	36.77	3.11	8.79
	RH36	15	3	1	24.66	9.53	7.15
	RH44	16	3	0	28.84	9.53	5.19
	RH47	16	4	1	34.43	9.53	8.02
	RH48	17	5	1	37.06	9.53	8.24
	RH49	17	5	1	26.59	9.53	7.59
	MDR1	10	1	4	19.58	3.11	3.44
	MDR3	17	5	1	30.01	9.29	5.41
	MDR4	17	4	0	29.29	9.91	5.03
	MDR33	12	2	3	19.14	9.53	4.25
	MDR36	15	4	2	40.26	9.53	10.41
RH	Control	14			17.38	9.91	3.58
	RH7	16	3	1	22.10	9.53	4.53
	RH12	17	4	1	42.50	9.91	8.70
	RH32	16	3	1	31.59	9.91	6.55
	RH34	15	3	2	36.38	11.06	8.07
	RH36	17	4	1	27.61	9.53	5.02
	RH44	13	1	2	21.80	9.53	5.46
	RH47	14	1	1	21.08	9.53	4.29
	RH48	13	0	1	18.90	9.53	4.49
	RH49	19	5	0	42.07	9.53	9.50
	MDR1	12	1	3	24.23	9.53	4.84
	MDR3	16	3	1	23.94	11.06	3.34
	MDR4	16	4	2	35.73	9.53	8.89
	MDR33	12	0	2	21.56	9.91	3.91
	MDR36	17	3	0	43.89	9.53	13.29

Figs 3 and 4 show the concentrations of HPLC-detected metabolites and the clustering of all of the treatments in this experiment based on the detected metabolite profiles. For CS grape leaves, 22 metabolites were detected, and the numbers of detected metabolites in grape leaves with different treatments varied from 9 to 17. The metabolite M24 only appeared in fungal strain MDR33 (*Colletotrichum* sp.)-infected CS grape leaves (Fig 3). Metabolites M3, M7, M10, M20 and M23 were detected with 2 to 4 fungal strains of treated CS leaves, but other metabolites were detected in more than 5 fungal strain-inoculated CS leaves. Fungal strain MDR36 initiated the highest concentration of metabolite M8 (10.41 mg/g) in CS grape leaves (Table 2, S2 Table). A higher content (>8 mg/g) of metabolite M8 was detected in fungal strain RH47 and RH48-treated CS grape leaves. The symbiosis of fungal strain RH34 also initiated a higher content (≥8 mg/g) metabolite M1 in CS leaves.

For RH grape leaves, 21 metabolites were detected, and the numbers of the detected metabolites in grape leaves with different treatments varied from 12 to 17 (Fig 4, Table 2). Metabolite M6 only appeared in fungal strain RH36 (*Fusarium* sp.)-treated grape leaves. Metabolites M3, M10 and M21 were detected in 2 or 4 fungal strain-treated grape leaves. Similarly, fungal strain MDR36 initiated the highest concentration of metabolite M8 (13.29 mg/g) in RH grape leaves (Table 2, S3 Table). The infection of endophytic fungal strains RH49 and MDR4 triggered higher concentrations (≥8 mg/g) of metabolite M8, and inoculation with fungal strains RH12 and RH34 also initiated higher concentrations (≥8 mg/g) of metabolites M9 and M11, respectively in RH grape leaves (S3 Table). Metabolites M23 and M24, which were detected in CS leaves, were not detected in RH leaves, and metabolite M18 was detected in RH leaves but not in CS leaves.

Clustering of the fungal strains used in this study based on the metabolite patterns revealed that all strains could be divided into 3 and 4 groups in RH and CS grape leaves, respectively (Figs 3 and 4). For CS grape leaves, group 1 included 9 fungal strains that were closely clustered with the control, which suggested the decreased metabolic impact on CS grape leaves (Fig 3). Except for the control, the fungal treatments in group 1 involved strains from the genera *Fusarium* (3/5, three of five used in this study), *Alternaria* (2/2), *Colletotrichum* (1/3), *Epicoccum* (1/1) and *Nigrospora* (2/2). Fungal strains MDR3 and MDR4 from genera *Fusarium* (2/5) clustered in group 2, and strains RH48 and MDR36 from genera *Colletotrichum* (2/3) clustered in group 3. The remaining group contained only RH34 (*Trichothecium*) and exhibited the strongest effect on CS leaf metabolomics. For RH grape leaves, group 1 included 8 strains that closely clustered with control, including strains from genera *Fusarium* (3/5), *Colletotrichum* (2/3), *Alternaria* (1/2), *Epicoccum* (1/1) and *Nigrospora* (1/2). Group 2 included RH32 (*Alternaria*), RH34 (*Trichothecium*) and RH12 (*Nigrospora*). RH49 (*Fusarium*), MDR4 (*Fusarium*) and MDR36 (*Colletotrichum*) clustered into group 3 and conferred the greatest effects on the metabolism of RH grape leaves (Fig 4).

CS grape leaves treated with fungal strains RH48, RH49, MDR3 and MDR4 produced the most metabolites (17), and RH34-treated CS grape leaves produced the least metabolites (9) (Fig 4). Compared to the control, 1 to 5 novel metabolites were introduced in CS grape leaves due to the symbiosis of fungal strains, except RH12 and RH34 (Table 2). Infection with fungal strains RH48, RH49 and MDR3 introduced the most numbers of novel metabolites (5) in CS leaves. In contrast, the symbiosis of fungal strains obviously suppressed the production of 1 to 4 metabolites, except RH44 and MDR4, compared to the basic metabolite profiles of CS grape leaves. RH34 and MDR1 suppressed the most metabolites (4). Treatment of RH grape leaves with RH12, RH36, RH49 and MDR36 lead to the detection of the most metabolites (17 or 19), and treatment with RH44, RH47, RH48, MDR1 and MDR33 produced the fewer numbers (12–14) of metabolites (Table 2). Cultivation with fungal strains produced 1 to 5 novel metabolites in RH leaves, except RH48 and MDR33, compared to the control. Fungal strain RH49 introduced the most number of novel metabolites (5) into RH grape leaves. Co-cultivation with fungal strains suppressed 1 to 3 metabolites compared to the basic metabolites of RH grape leaves, except RH49 and MDR36. MDR1 suppressed the most metabolites (3) in RH grape leaves.

Overall, fungal strain RH49 initiated the most numbers of metabolites and introduced the greatest number of novel metabolites in CS and RH grape leaves (Table 2). The fewest numbers of metabolites were detected in grape leaves treated with MDR1 and MDR33. Fewer novel metabolites were detected in MDR1-treated leaves, and MDR1 suppressed the most metabolites in CS and RH grape leaves. Strain MDR36 initiated greater effects on grape metabolites in CS and RH leaves, especially metabolite M8, which reached the maximum concentration of the detected metabolites in CS and RH grape leaves (10.41 mg/g and 13.29 mg/g, respectively) (S2 and S3 Tables).

In addition to the qualitative shaping of fungal endophytes on the metabolite profiles of grape leaves, quantitative effects on metabolites codetected in all treatments were observed (Tables 3 and 4). Nine metabolites were codetected in all CS and RH leaf samples, and 8 of these metabolites were codetected in both varieties. Metabolite M15 was only detected in CS leaves, and metabolite M5 was only detected in RH leaves. The concentrations of these metabolites and the different significances between treatments are presented in Tables 3 and 4. The concentration of these metabolites in grape leaves were differentially influenced by the symbiosis of diverse fungal strains, and some of these changes reached statistical significance (Tables 3 and 4). For CS grape leaves, RH34, RH36 and MDR1 significantly promoted the contents of all the co-detected metabolites compared to the control (Table 3). The symbioses of fungal strains RH12, RH36, RH49, MDR4 and MDR36 significantly promoted the concentrations of all of these co-detected metabolites in RH leaves (Table 4). Metabolite G at a retention time of 11.32 min was greatly increased by all of the fungal strains in RH grape leaves (Table 4).

**Table 3:** Content of co-detected metabolites in CS grape leaves and the different significances.

Compound Treatment	M1 (RT = 3.11)	M2 (RT = 3.52)	M4 (RT = 8.48)	M8 (RT = 9.53)	M9 (RT = 9.91)	M11 (RT = 11.06)	M12 (RT = 11.32)	M13 (RT = 12.53)	M15 (RT = 13.19)
Control	1.12±0.02	1.24±0.07	0.38±0.06	2.03±0.01	3.53±0.02	1.69±0.04	0.92±0.03	1.71±0.06	1.59±0.06
RH7	1.18±0.04	1.44±0.07**	0.48±0.01	3.37±0.12**	5.55±0.28**	3.23±0.03**	1.08±0.01	0.94±0.01**	0.77±0.14**
RH12	1.57±0.01**	1.49±0.01**	0.25±0.02	2.11±0.01	2.69±0.49**	4.01±0.07**	3.77±0.09**	1.74±0.07	2.78±0.13**
RH32	1.11±0.01	2.04±0.03**	0.42±0.01	2.79±0.02**	2.38±0.01**	3.81±0.09**	2.81±0.08**	0.96±0.04**	0.78±0.08**
RH34	8.79±0.11**	3.26±0.09**	2.74±0.06**	3.78±0.11**	4.28±0.40**	5.53±0.32**	2.13±0.17**	3.78±0.27**	2.48±0.08**
RH36	0.97±0.01**	1.80±0.03**	0.64±0.00**	7.15±0.03**	4.16±0.08*	3.54±0.22**	1.75±0.06**	0.87±0.03**	0.53±0.01**
RH44	1.61±0.01**	2.45±0.02**	0.54±0.02	5.19±0.04**	3.08±0.07	3.37±0.09**	3.26±0.10**	1.60±0.02	1.69±0.01
RH47	1.24±0.03	1.67±0.06**	0.67±0.02**	8.02±0.01**	6.34±0.04**	4.33±0.12**	4.74±0.17**	1.45±0.02*	1.31±0.02*
RH48	1.46±0.07**	1.88±0.05**	5.72±0.10**	8.24±0.21**	3.39±0.25	5.57±0.63**	1.56±0.06**	2.04±0.02**	2.06±0.02**
RH49	1.11±0.04	1.68±0.04**	0.30±0.00	7.59±0.09**	4.76±0.02**	2.18±0.08	1.83±0.04**	1.12±0.01**	2.15±0.01**
MDR1	3.44±0.04**	1.38±0.03*	1.87±0.16**	2.47±0.15**	2.43±0.17**	2.96±0.13**	1.82±0.20**	1.42±0.10**	1.07±0.04**
MDR3	0.88±0.01**	1.23±0.02	0.39±0.02	2.82±0.06**	4.97±0.05**	3.94±0.95**	0.91±0.10	1.33±0.00**	1.31±0.17*
MDR4	1.21±0.00	1.66±0.01**	0.31±0.01	2.93±0.08**	5.03±0.01**	2.84±0.04**	2.43±0.04**	2.64±0.01**	2.27±0.04**
MDR33	0.97±0.07**	0.51±0.01**	0.42±0.01	4.25±0.01**	4.03±0.03	1.77±0.02	1.24±0.01*	2.09±0.04**	1.64±0.04
MDR36	2.27±0.02**	1.26±0.03	1.56±0.10**	10.41±0.09**	3.78±0.18	6.46±0.31**	4.34±0.12**	2.92±0.02**	1.41±0.04

Values were indicated as ‘mean ± standard errors’ with different significances marked as‘*’ or ‘**’, compared to the control. *: significant difference at 5%, and **: significant difference at 1% (Tukey’s Test).

**Table 4:** Content of co-detected metabolites in RH grape leaves and the different significances.

Compound Treatment	M1 (RT = 3.11)	M2 (RT = 3.52)	M4 (RT = 8.48)	M5 (RT = 8.67)	M8 (RT = 9.53)	M9 (RT = 9.91)	M11 (RT = 11.06)	M12 (RT = 11.32)	M13 (RT = 12.53)
Control	0.95±0.01	1.59±0.06	0.47±0.02	0.43±0.01	3.04±0.01	3.58±0.04	1.72±0.05	0.56±0.02	0.84±0.01
RH7	1.16±0.01	1.76±0.03*	0.53±0.01	0.46±0.02	4.53±0.01**	3.81±0.01	2.27±0.01**	1.55±0.02**	0.49±0.02**
RH12	1.74±0.02**	2.16±0.06**	2.21±0.02**	1.42±0.03**	5.80±0.13**	8.70±0.44**	5.98±0.25**	2.33±0.01**	1.91±0.02**
RH32	1.42±0.01**	1.25±0.02**	0.59±0.01*	0.51±0.01	3.48±0.02**	6.55±0.16**	6.11±0.33**	3.06±0.16**	1.45±0.04**
RH34	1.76±0.02**	1.18±0.04**	0.50±0.01	0.56±0.01**	5.95±0.10**	7.00±0.08**	8.07±0.05**	3.40±0.09**	1.49±0.02**
RH36	1.73±0.00**	2.36±0.10**	1.18±0.02**	1.05±0.04**	5.02±0.07**	2.23±0.05**	4.50±0.17**	2.40±0.17**	1.65±0.01**
RH44	1.28±0.02**	1.91±0.05**	0.56±0.01	0.36±0.02	5.46±0.09**	3.82±0.04	2.10±0.02*	1.54±0.05**	1.01±0.20
RH47	1.49±0.00**	2.22±0.01**	0.57±0.04	0.61±0.08**	4.29±0.10**	3.09±0.02**	2.09±0.06*	2.01±0.05**	0.89±0.02
RH48	1.11±0.02	1.66±0.04	0.51±0.01	0.27±0.00**	4.49±0.03**	3.51±0.10	1.85±0.02	1.22±0.02**	0.89±0.01
RH49	2.01±0.02**	1.44±0.01*	1.54±0.11**	1.69±0.01**	9.50±0.06**	4.48±0.03**	5.36±0.03**	3.90±0.09**	2.01±0.04**
MDR1	3.65±0.33**	2.37±0.06**	1.26±0.03**	0.63±0.00**	4.84±0.02**	3.55±0.04	2.40±0.04**	1.36±0.06**	1.27±0.03**
MDR3	1.44±0.05**	1.68±0.01	0.63±0.01**	0.89±0.06**	3.12±0.02	2.35±0.03**	3.34±0.03**	1.98±0.01**	1.48±0.01**
MDR4	1.24±0.01**	1.93±0.06**	1.21±0.07**	1.06±0.07**	8.89±0.12**	4.92±0.21**	3.30±0.02**	2.83±0.13**	1.58±0.02**
MDR33	1.11±0.02	1.59±0.02	0.53±0.02	0.55±0.03**	3.77±0.02**	3.91±0.04	2.23±0.00**	1.01±0.01**	1.35±0.05**
MDR36	1.65±0.03**	1.97±0.03**	1.94±0.06**	1.78±0.02**	13.29±0.02**	5.27±0.01**	5.43±0.04**	3.01±0.26**	1.70±0.03**

**Fig 5:**
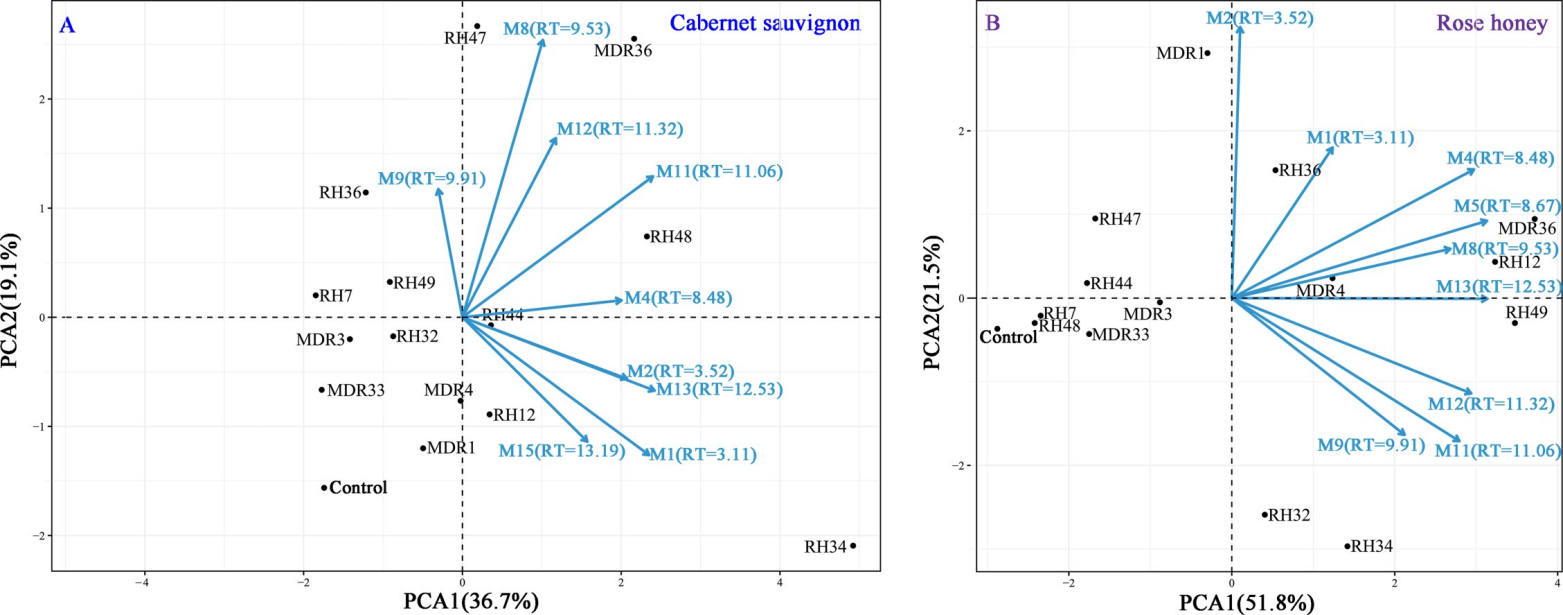
PCA of the impact of endophytic fungal infection on codetected metabolites in CS and RH leaves.

To visualize the effects of endophytic fungi inoculation on the co-detected metabolites, principal component analysis (PCA) was performed using R package (Fig 5). The principal components of CS (PC1, PC2) explained 36.7% and 19.1% of the total variances, respectively (Fig 5A). In contrast, the principal components of RH (PC1, PC2) explained 51.8% and 21.5% of the total variances, respectively (Fig 5B). PCA provided a visual representation of the impact of endophytic fungi inoculation on the co-detected metabolites. For CS leaves, strains RH34, RH48 and MDR36 primarily contributed positively to all of the co-detected metabolites, except M9. Strains RH12, RH49, MDR4 and MDR36 primarily contributed positively to all of the co-detected metabolites for RH leaves.

To illustrate whether the strains that have a similar effect for the grapevines have similar metabolites patterns, we also detected the metabolites profiles of fungal endophytes by HPLC assay with the same conditions as the analysis of grape leaves (S4 Table; S5 and S6 Figs). From heat-map and clustering of HPLC detected metabolites contents in fungal endophytes (S6 Fig), we learned the strains from the same genus had similar metabolites patterns and tended to be clustered together. The numbers of detected metabolites in fungal endophytes varied from 5 to 13, and metabolites at retention time of 3.11, 9.53, 9.91 and 16.19 min were codetected in all strains. And 7 metabolites at retention time of 3.11, 3.52, 9.53, 9.91, 13.51, 14.54 and 16.19 min detected in some of strains were also detected in leaves samples (metabolites as M1, M2, M8, M9, M16, M18 and M21). Fungal strains RH48 produced the most number of metabolites (13 metabolites), whereas in strains RH7 and RH36 detected the least number of metabolites (5), and RH34, RH44, MDR1, MDR3, MDR36 produced 11 or 9 metabolites.

## Discussion

Numerous and diverse species of fungal endophytes systematically colonize plants [2, 22]. Because endophytes may biochemically contribute to the vegetative parts (such as leaves) and the fruits of host plants, the interactions between fungal endophytes and the host plant deserve a thorough investigation. The beneficial effects of some endophytes on host plants were developed as plant growth promoters, biocontrol agents, and producers of novel secondary metabolites [23–26]. However, relationships between endophytes and their host plants are far from understood. Only a few studies examined the biochemical effects of fungal endophytes on grapes and the resultant wines. These fungal endophytes have the capacity to produce plant secondary metabolites, especially resveratrol, trans-resveratrol and its oligomer, which alter the grape metabolite composition [27–29]. Recent works demonstrated the metabolic impact of endophytes on grape cells [30, 31], but no reports examined these issues on vine leaves. The inoculation of eight fungal endophytes isolated from *Vitis vinifera* exerted significant effects on the physio-chemical status of field-grown grapevines [32]. However, due to the complex endophytic communities within grapevines *in vivo*, it is difficult to identify the metabolic functions of certain endophytes. Therefore, the present study used *in vitro* grape leaves to establish the leaf-endophyte symbionts and analyzed the effects of the symbiosis of certain endophytic fungal strains on the metabolic profiles of grape leaves. Similar approaches were used for the rapid screening of germplasms for resistance to bacterial pathogens [33, 34], and this method was used to evaluate the host range and virulence for citrus bacterial diseases [35, 36].

To predict the metabolites in the extracts, we performed preliminary experiments on the extracts of CS leaves treated with strain RH44, with catechin added as a reference molecule. We found that the peak height and peak area showed huge improvements in the metabolite at a retention time of approximately 11 min (S7 Fig). The retention time was basically the same as that of the extract of strain RH44-treated CS leaves and the reference catechin, which indicated that the metabolite that was observed at a retention time of 11 min was catechin. Based on the metabolite extraction and HPLC elution, together with the results of numerous previous studies [37–44], we speculated that the analyzed metabolites were organic acids (such as caffeic acid, syringic acid, and gallic acid), proanthocyanins, flavanols (such as rutin, kaempferol, and quercetin), and flavan-3-ols (such as epicatechin, epigallocatechin, and catechin). These metabolites play an important role in grape flavor and the resultant wine quality. Therefore, the reconstruction of endophytic fungal populations of hosts would likely contribute to the biochemical composition and further influence the quality of the final product.

As expected, the contents and composition of the detected metabolites were fundamentally different in CS and RH grape leaves (Figs 3 and 4). The detected number of metabolites in CS and RH grape leaves without endophytic fungi inoculations were not greatly different, but infection with the same batch of endophytic fungi obviously triggered more metabolite responses in CS than RH grape leaves. Notably, the metabolic patterns of grape leaves shaped by fungal endophytes exhibited fungal strain-specificity in different grape cultivars (Figs 3 and 4). Samples of CS and RH grape leaves infected with RH49 (*Fusarium* sp.) and MDR36 (*Colletotrichum* sp.) contained the highest counts of total metabolites and greater counts of novel metabolites. MDR36-treated samples contained the maximum total contents of the detected metabolites in CS and RH leaves. Treatments with MDR1 (*Nigrospora* sp.) and MDR33 (*Colletotrichum* sp.) produced the lowest numbers of total metabolites and novel metabolites and greatly suppressed the metabolites in CS and RH leaves (Table 2). Notably, strain RH49 in CS and RH leaves exhibited stronger symbiosis and triggered a greater response of the detected metabolites, and strain MDR1 in CS and RH leaves suppressed the detected metabolites despite stronger symbiosis.

Mechanisms underlying the metabolic impact of endophytes on the host plant included: endophytes self-metabolizing, endophytes and host co-metabolizing, and signaling [45]. In our study, 5 metabolites at retention time of 3.11, 3.52, 9.53, 9.91 and 13.51 min detected in some of strains were also detected in leaves samples including control sample (metabolites as M1, M2, M8, M9 and M16), which gave a hint that a mutualistic and symbiotic relationship was gradually set up between endophytes and plants such as co-metabolizing pathways during the long period of co-evolution. Two metabolites at retention time of 14.54 and 16.19 min were detected in fungal endophytes and some of CS or RH samples treated with strains, but not detected in control leaves sample, which suggested that the metabolites were introduced by the symbiosis of fungal endophytes.

With all the experimental results, we came to the conclusion that fungal endophytes inoculation could reshaped the metabolic profiles of grape leaves of both cultivars by self-metabolizing and co-metabolizing pathways, and this modification appeared obvious strain-specificities. Consistent with previous studies, Yu et al. [46] showed that exposure to fungal endophytes could quantitatively and compositionally modify the anthocyanins in grape cells. Our results confirmed the successful symbiosis of fungal endophytes, and the symbiosis triggered physio-biochemical responses and metabolic profiles, which support the use endophytic fungi to improve the quality of crops, such as coffee, tea or wine grapes.

## Supporting information

S1 Table Acetonitrile-water gradient for methanol extracts of grape leaves separation and analysis on reversed-phase HPLC. (DOCX)

S2 Table HPLC detected metabolites and content of CS grape leaves (mg/g). (DOCX)

S3 Table HPLC detected metabolites and content of RH grape leaves (mg/g). (DOCX)

S4 Table HPLC detected metabolites and content of fungal endophytes (mg/g). (DOCX)

S1 Fig Chromatograms of CS grape leaf extracts after infection with different endophytic fungal strains. (TIF)

S2 Fig Chromatograms of RH grape leaf extracts after infection with different endophytic fungal strains. (TIF)

S3 Fig Clustering of replicates of the treatments of CS based on the appearance and absence of detected metabolites using Squared Euclidean distance Hierarchical clustering in SPSS 16.0 software. (TIF)

S4 Fig Clustering of replicates of the treatments of RH based on the appearance and absence of detected metabolites using Squared Euclidean distance Hierarchical clustering in SPSS 16.0 software. (TIF)

S5 Fig Chromatograms of different fungal endophytes. (TIF)

S6 Fig Heat map and clustering of HPLC detected metabolites contents in fungal endophytes. (TIF)

S7 Fig The preliminary experiment to evaluate the presence of catechin in CS leaves treated with strain RH44. (TIF)
