# Supplementary material for: The symbioses of endophytic fungi shaped the metabolic profiles in grape leaves of different varieties
Source: PLoS One. 2020 Sep 11;15(9):e0238734. doi: 10.1371/journal.pone.0238734 (PMC7485881; doi:10.1371/journal.pone.0238734)
Supplement: S1 Table — (DOCX) [file pone.0238734.s001.docx]

**S1 Table.** Acetonitrile-water gradient for methanol extracts of grape leaves separation and analysis on reversed-phase HPLC.

| Time (min) | Flow (mL/min) | % ACN | % Water (mixed with methanol) |
| --- | --- | --- | --- |
| 0 | 1 | 95 | 5 |
| 2.0 | 1 | 95 | 5 |
| 2.1 | 1 | 90 | 10 |
| 5.0 | 1 | 90 | 10 |
| 5.1 | 1 | 85 | 15 |
| 9.0 | 1 | 85 | 15 |
| 9.1 | 1 | 80 | 20 |
| 15.0 | 1 | 80 | 20 |
| 15.1 | 1 | 95 | 5 |
| 20.0 | 1 | 95 | 5 |
