# Supplementary material for: The symbioses of endophytic fungi shaped the metabolic profiles in grape leaves of different varieties
Source: PLoS One. 2020 Sep 11;15(9):e0238734. doi: 10.1371/journal.pone.0238734 (PMC7485881; doi:10.1371/journal.pone.0238734)
Supplement: S2 Table — (DOCX) [file pone.0238734.s002.docx]

**S2 Table.** HPLC detected metabolites and content of CS grape leaves (mg/g).

| **RT**  **T** | 3.11 | 3.52 | 4.50 | 5.75 | 8.48 | 8.67 | 9.08 | 9.29 | 9.53 | 9.91 | 10.76 | 11.06 | 11.32 | 12.53 | 12.89 | 13.19 | 13.51 | 14.05 | 14.54 | 15.20 | 15.72 | 16.19 | 16.71 | 17.14 | 17.88 |
| --- | --- | --- | --- | --- | --- | --- | --- | --- | --- | --- | --- | --- | --- | --- | --- | --- | --- | --- | --- | --- | --- | --- | --- | --- | --- |
| Control | 1.12 | 1.24 | - | - | 0.38 | - | 0.68 | 1.26 | 2.03 | 3.53 | - | 1.69 | 0.92 | 1.71 | - | 1.59 | 1.06 | 0.26 | - | - | - | - | - | - | - |
| RH7 | 1.18 | 1.44 | - | - | 0.48 | 0.61 | 1.30 | - | 3.37 | 5.55 | - | 3.23 | 1.08 | 0.94 | - | 0.77 | 0.42 | 0.37 | - | - | - | - | - | - | - |
| RH12 | 1.57 | 1.49 | - | - | 0.25 | - | 1.30 | - | 2.11 | 2.69 | - | 4.01 | 3.77 | 1.74 | - | 2.78 | 0.60 | - | - | - | - | - | - | - | - |
| RH32 | 1.11 | 2.04 | - | - | 0.42 | 0.30 | 1.24 | - | 2.79 | 2.38 | - | 3.81 | 2.81 | 0.96 | 0.59 | 0.78 | 0.36 | 0.37 | - | - | - | - | 0.42 | - | - |
| RH34 | 8.79 | 3.26 | - | - | 2.74 | - | - | - | 3.78 | 4.28 | - | 5.53 | 2.13 | 3.78 | - | 2.48 | - | - | - | - | - | - | - | - | - |
| RH36 | 0.97 | 1.80 | - | - | 0.64 | 0.45 | 0.46 | - | 7.15 | 4.16 | - | 3.54 | 1.75 | 0.87 | - | 0.53 | 0.47 | 0.60 | - | 0.44 | - | - | 0.81 | - | - |
| RH44 | 1.61 | 2.45 | 0.77 | - | 0.54 | - | 1.98 | 1.01 | 5.19 | 3.08 | - | 3.37 | 3.26 | 1.60 | 0.83 | 1.69 | 0.57 | 0.37 | - | - | - | - | 0.51 | - | - |
| RH47 | 1.24 | 1.67 | 0.46 | - | 0.67 | - | 1.46 | - | 8.02 | 6.34 | - | 4.32 | 4.74 | 1.45 | 0.71 | 1.31 | 0.74 | 0.37 | - | 0.35 | - | - | 0.58 | - | - |
| RH48 | 1.46 | 1.88 | - | - | 5.72 | 0.73 | 0.79 | - | 8.24 | 3.39 | 0.34 | 5.57 | 1.56 | 2.03 | 1.05 | 2.06 | 0.58 | 0.44 | - | 0.64 | - | - | 0.57 | - | - |
| RH49 | 1.11 | 1.68 | 0.49 | - | 0.30 | 0.34 | 0.53 | - | 7.59 | 4.76 | - | 2.18 | 1.83 | 1.12 | 0.58 | 2.15 | 0.91 | 0.27 | - | 0.28 | - | - | 0.48 | - | - |
| MDR1 | 3.44 | 1.38 | - | - | 1.87 | - | - | - | 2.47 | 2.43 | - | 2.96 | 1.82 | 1.42 | - | 1.07 | - | - | - | - | - | - | 0.72 | - | - |
| MDR3 | 0.88 | 1.23 | - | - | 0.39 | 0.32 | - | 5.41 | 2.82 | 4.97 | - | 3.94 | 0.91 | 1.33 | 1.03 | 1.31 | 1.00 | 0.68 | - | - | 0.57 | - | 1.11 | 2.11 | - |
| MDR4 | 1.21 | 1.66 | - | - | 0.31 | 0.39 | 0.39 | 3.84 | 2.93 | 5.03 | - | 2.84 | 2.43 | 2.64 | - | 2.27 | 1.17 | 0.53 | - | 0.32 | - | - | 0.82 | 0.52 | - |
| MDR33 | 0.97 | 0.51 | - | - | 0.42 | 0.48 | - | - | 4.25 | 4.03 | - | 1.77 | 1.24 | 2.09 | - | 1.64 | 1.10 | - | - | - | - | - | - | - | 0.64 |
| MDR36 | 2.27 | 1.26 | - | - | 1.56 | 1.50 | - | - | 10.41 | 3.78 | 1.45 | 6.46 | 4.34 | 2.92 | - | 1.41 | 0.82 | 0.59 | - | - | 0.99 | - | 0.49 | - | - |
