# Supplementary material for: The symbioses of endophytic fungi shaped the metabolic profiles in grape leaves of different varieties
Source: PLoS One. 2020 Sep 11;15(9):e0238734. doi: 10.1371/journal.pone.0238734 (PMC7485881; doi:10.1371/journal.pone.0238734)
Supplement: S3 Table — (DOCX) [file pone.0238734.s003.docx]

**S3 Table.** HPLC detected metabolites and content of RH grape leaves (mg/g).

| **RT**  **T** | 3.11 | 3.52 | 4.50 | 5.75 | 8.48 | 8.67 | 9.08 | 9.29 | 9.53 | 9.91 | 10.76 | 11.06 | 11.32 | 12.53 | 12.89 | 13.19 | 13.51 | 14.05 | 14.54 | 15.20 | 15.72 | 16.19 | 16.71 | 17.14 | 17.88 |
| --- | --- | --- | --- | --- | --- | --- | --- | --- | --- | --- | --- | --- | --- | --- | --- | --- | --- | --- | --- | --- | --- | --- | --- | --- | --- |
| Control | 0.95 | 1.59 | - | - | 0.47 | 0.43 | - | 1.50 | 3.04 | 3.58 | - | 1.72 | 0.56 | 0.84 | 0.21 | 1.52 | 0.67 | 0.30 | - | - | - | - | - | - | - |
| RH7 | 1.16 | 1.76 | 0.48 | - | 0.53 | 0.46 | - | 1.57 | 4.53 | 3.81 | - | 2.27 | 1.55 | 0.49 | 0.44 | 1.50 | 0.98 | - | - | 0.33 | - | - | 0.25 | - | - |
| RH12 | 1.74 | 2.16 | - | - | 2.21 | 1.42 | - | 1.40 | 5.80 | 8.70 | 1.54 | 5.98 | 2.33 | 1.91 | - | 2.71 | 0.78 | 0.48 | 0.55 | 1.73 | - | 1.06 | - | - | - |
| RH32 | 1.42 | 1.25 | - | - | 0.59 | 0.51 | - | 1.22 | 3.48 | 6.55 | - | 6.11 | 3.06 | 1.45 | - | 1.49 | 0.59 | 0.34 | - | 1.75 | - | 1.29 | 0.48 | - | - |
| RH34 | 1.76 | 1.18 | - | - | 0.50 | 0.56 | - | 2.18 | 5.95 | 7.00 | - | 8.07 | 3.40 | 1.49 | - | 2.52 | 0.47 | - | 0.26 | 0.56 | - | - | 0.49 | - | - |
| RH36 | 1.73 | 2.36 | - | - | 1.18 | 1.05 | 0.63 | 1.03 | 5.02 | 2.23 | - | 4.49 | 2.40 | 1.65 | 0.80 | 0.81 | 0.35 | - | 0.56 | 0.64 | - | - | 0.69 | - | - |
| RH44 | 1.28 | 1.91 | - | - | 0.56 | 0.36 | - | 0.54 | 5.46 | 3.82 | - | 2.10 | 1.54 | 1.01 | - | 0.99 | 1.73 | - | - | - | - | - | 0.49 | - | - |
| RH47 | 1.49 | 2.22 | - | - | 0.56 | 0.61 | - | 1.78 | 4.29 | 3.09 | - | 2.09 | 2.01 | 0.89 | 0.28 | 0.98 | 0.42 | - | - | - | - | - | 0.39 | - | - |
| RH48 | 1.11 | 1.66 | - | - | 0.51 | 0.27 | - | 1.54 | 4.49 | 3.51 | - | 1.85 | 1.22 | 0.89 | 0.29 | 1.02 | 0.53 | - | - | - | - | - | - | - | - |
| RH49 | 2.01 | 1.44 | 0.84 | - | 1.54 | 1.69 | - | 1.46 | 9.50 | 4.48 | 1.22 | 5.36 | 3.90 | 2.01 | 0.40 | 2.65 | 0.76 | 0.27 | 0.75 | 0.96 | - | - | 0.82 | - | - |
| MDR1 | 3.65 | 2.37 | - | - | 1.26 | 0.63 | - | - | 4.84 | 3.55 | - | 2.40 | 1.36 | 1.27 | 1.19 | - | - | 0.45 | - | - | - | - | 1.27 | - | - |
| MDR3 | 1.43 | 1.68 | - | - | 0.63 | 0.89 | - | 3.22 | 3.12 | 2.35 | - | 3.34 | 1.98 | 1.48 | 1.10 | - | 0.49 | 0.28 | 0.50 | 0.82 | - | 0.61 | - | - | - |
| MDR4 | 1.24 | 1.93 | 0.44 | - | 1.21 | 1.06 | - | 2.61 | 8.89 | 4.92 | - | 3.30 | 2.83 | 1.58 | - | 3.49 | 1.18 | - | 0.21 | 0.44 | - | - | 0.41 | - | - |
| MDR33 | 1.11 | 1.59 | - | - | 0.53 | 0.55 | - | 1.52 | 3.77 | 3.91 | - | 2.23 | 1.01 | 1.35 | - | 2.66 | 1.32 | - | - | - | - | - | - | - | - |
| MDR36 | 1.65 | 1.97 | 0.61 | - | 1.94 | 1.78 | - | 1.77 | 13.29 | 5.27 | - | 5.43 | 3.01 | 1.70 | 0.65 | 1.65 | 0.92 | 0.71 | - | 0.51 | - | - | 1.05 | - | - |
