# Supplementary material for: The symbioses of endophytic fungi shaped the metabolic profiles in grape leaves of different varieties
Source: PLoS One. 2020 Sep 11;15(9):e0238734. doi: 10.1371/journal.pone.0238734 (PMC7485881; doi:10.1371/journal.pone.0238734)
Supplement: S4 Table — (DOCX) [file pone.0238734.s004.docx]

**S4 Table.** HPLC detected metabolites and content of fungal endophytes (mg/g).

| **RT**  **T** | 3.11 | 3.39 | 3.52 | 4.14 | 5.75 | 7.55 | 8.01 | 9.53 | 9.91 | 10.22 | 12.07 | 13.51 | 14.554 | 16.19 |
| --- | --- | --- | --- | --- | --- | --- | --- | --- | --- | --- | --- | --- | --- | --- |
| RH7 | 2.15 | - | - | - | - | 1.86 | - | 5.01 | 6.49 | - | - | - | - | 5.15 |
| RH12 | 1.68 | - | - | 16.69 | 2.01 | - | - | 5.28 | 5.50 | 23.01 | - | - | - | 1.98 |
| RH32 | 3.07 | - | 2.25 | 64.57 | 1.92 | - | - | 9.81 | 12.05 | 89.48 | - | - | - | 9.51 |
| RH34 | 4.04 | - | 1.46 | 2.07 | 9.40 | - | - | 13.78 | 15.02 | 2.61 | 3.30 | 1.64 | 3.52 | 15.57 |
| RH36 | 2.10 | - | 1.18 | - | - | - | - | 11.05 | 17.69 | - | - | - | - | 15.33 |
| RH44 | 2.81 | - | 1.20 | 8.64 |  | - | - | 5.84 | 5.84 | 23.33 | - | - | - | 2.82 |
| RH47 | 1.34 | - | - | - | 9.12 | 2.03 | - | 7.31 | 10.19 | 2.30 | 3.28 | - | 1.51 | 5.17 |
| RH48 | 2.54 | 2.09 | 1.16 | - | 1.92 | 1.84 | 1.66 | 12.41 | 10.12 | 1.82 | 2.06 | 1.30 | 4.19 | 10.32 |
| RH49 | 2.35 | - | - | - | 2.06 | - | - | 11.64 | 17.37 | 1.22 | - | - | 1.10 | 11.32 |
| MDR1 | 2.82 | 1.52 | 1.41 | - | 4.65 | 2.22 | - | 4.75 | 15.44 | - | - | - | 1.76 | 6.35 |
| MDR3 | 2.65 | 2.90 | - | - | 9.13 | - | - | 8.59 | 3.25 | 11.18 | 4.45 | - | 3.95 | 6.73 |
| MDR4 | 2.51 | - | - | - | 11.90 | - | - | 2.06 | 1.83 | 7.17 | - | - | 6.89 | 4.17 |
| MDR33 | 2.87 | - | - | - | 13.46 | - | - | 6.87 | 4.27 | 1.92 | 4.66 | - | 6.38 | 12.20 |
| MDR36 | 3.77 | - | 8.57 | 8.06 | 2.58 | - | - | 6.70 | 4.56 | 5.53 | - | - | 2.32 | 6.81 |
