# Supplementary figures and images for: The symbioses of endophytic fungi shaped the metabolic profiles in grape leaves of different varieties
Source: PLoS One. 2020 Sep 11;15(9):e0238734. doi: 10.1371/journal.pone.0238734 (PMC7485881; doi:10.1371/journal.pone.0238734)

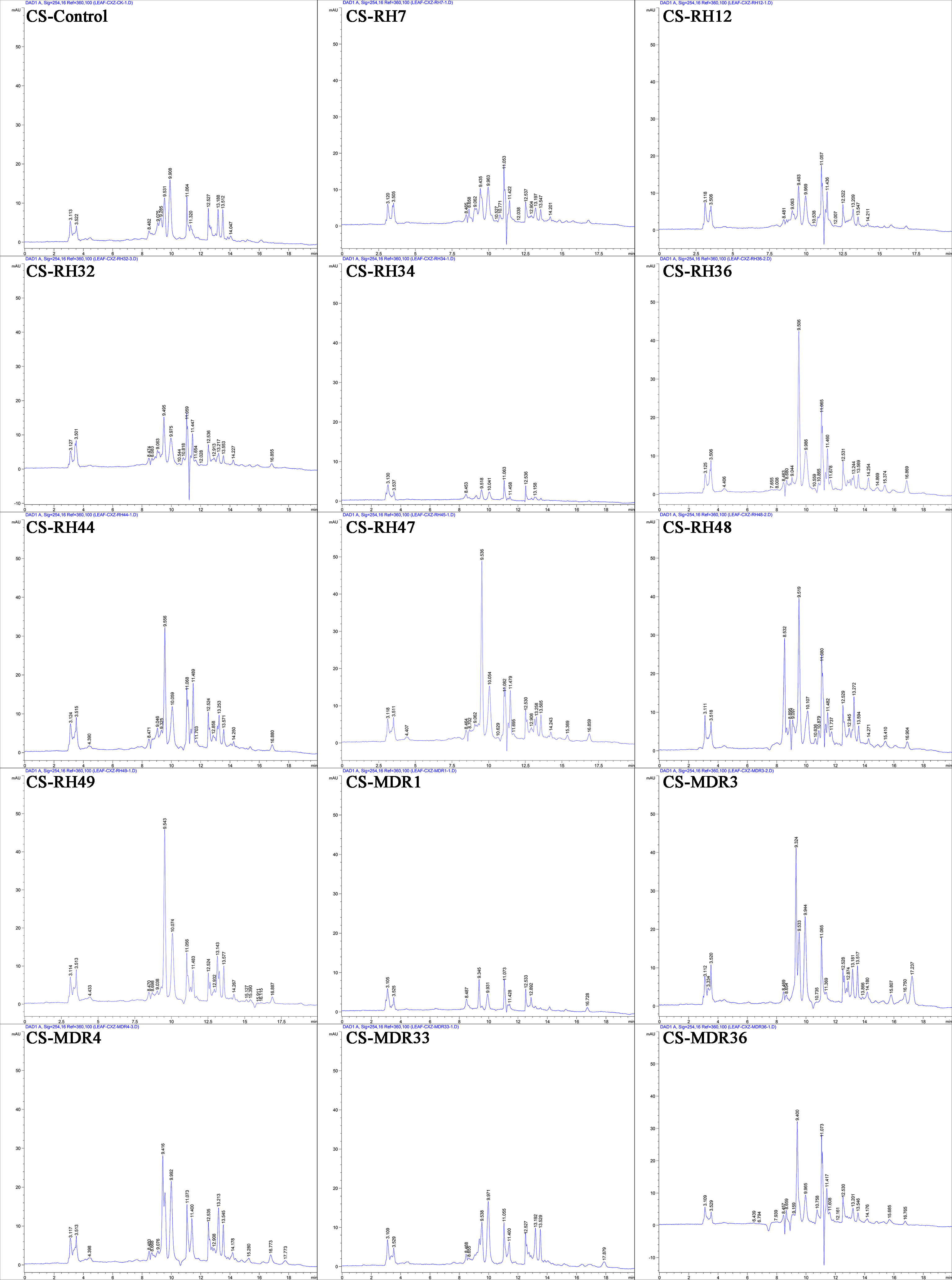

Supplement: S1 Fig — (TIF) [file pone.0238734.s005.tif]

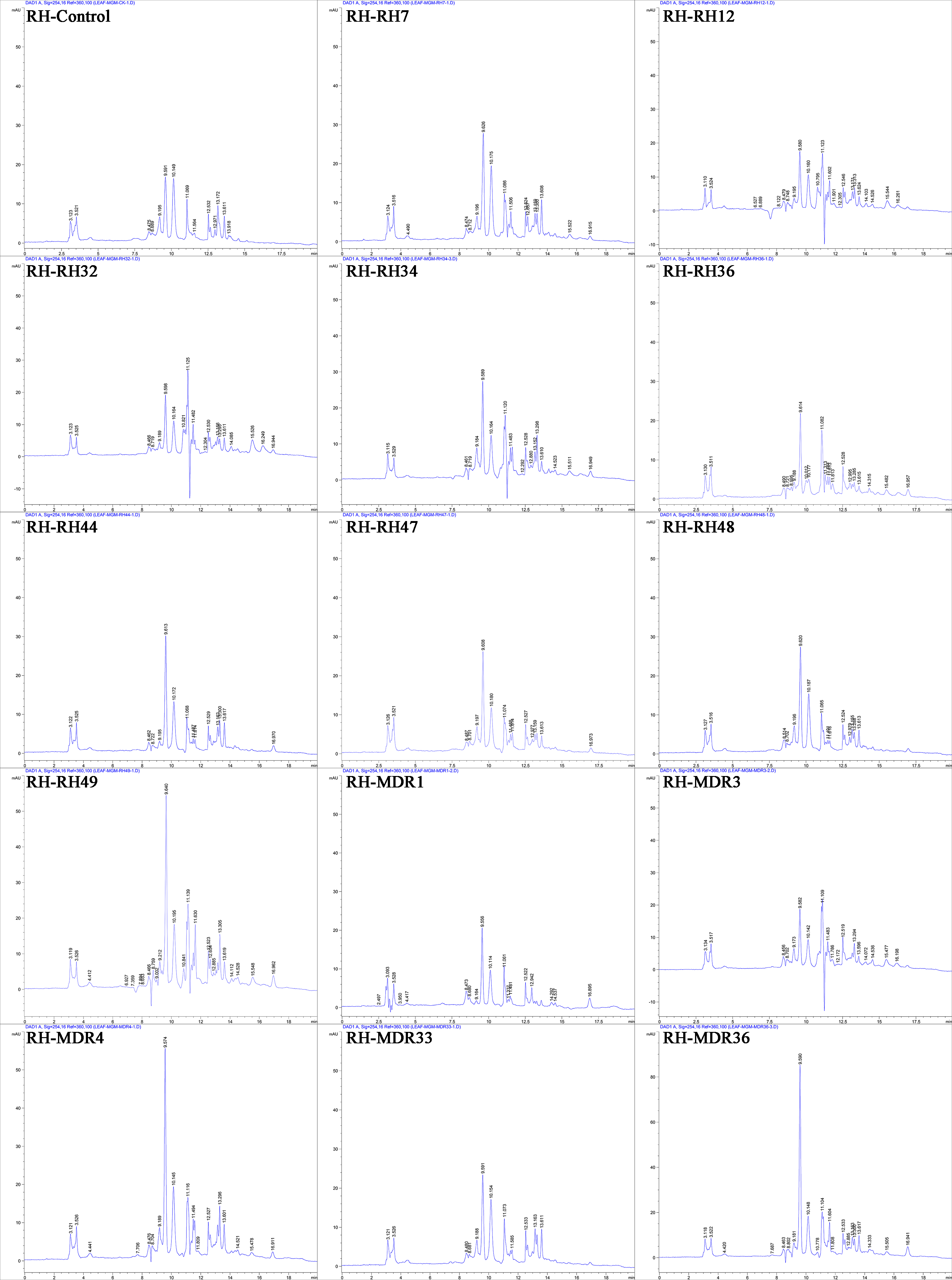

Supplement: S2 Fig — (TIF) [file pone.0238734.s006.tif]

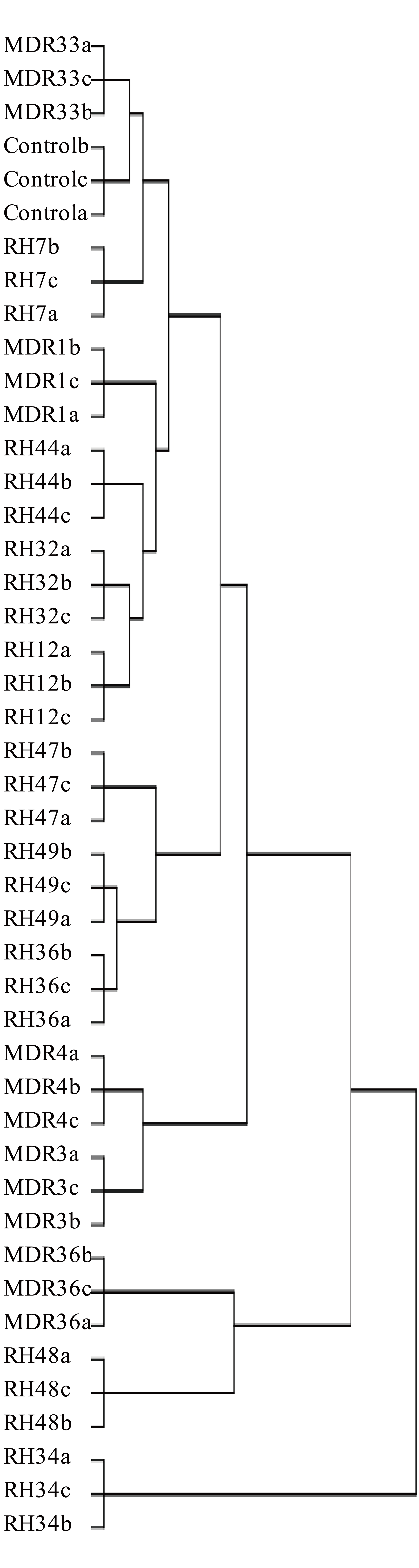

Supplement: S3 Fig — (TIF) [file pone.0238734.s007.tif]

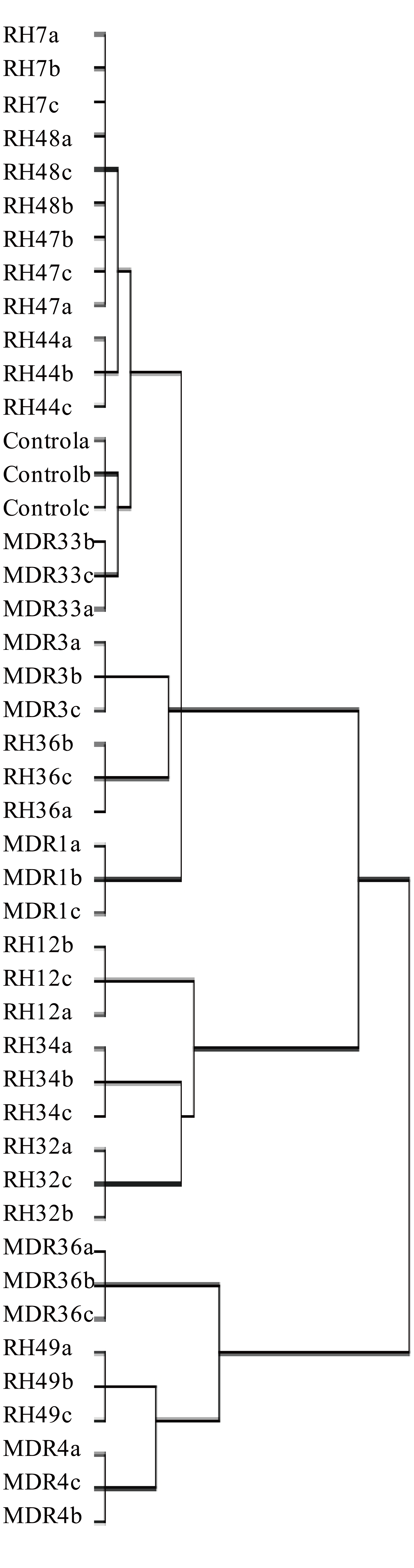

Supplement: S4 Fig — (TIF) [file pone.0238734.s008.tif]

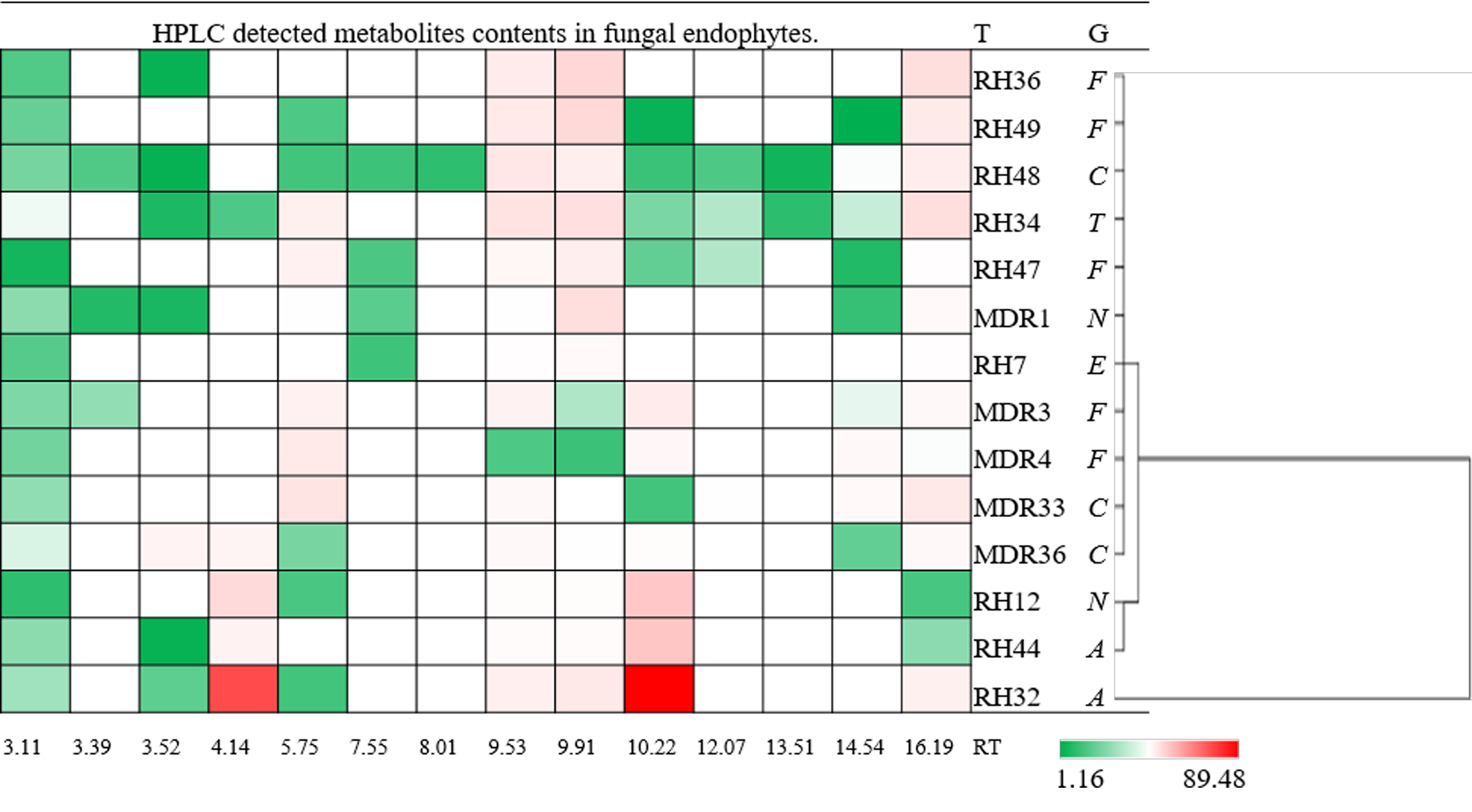

Supplement: S6 Fig — (TIF) [file pone.0238734.s010.tif]

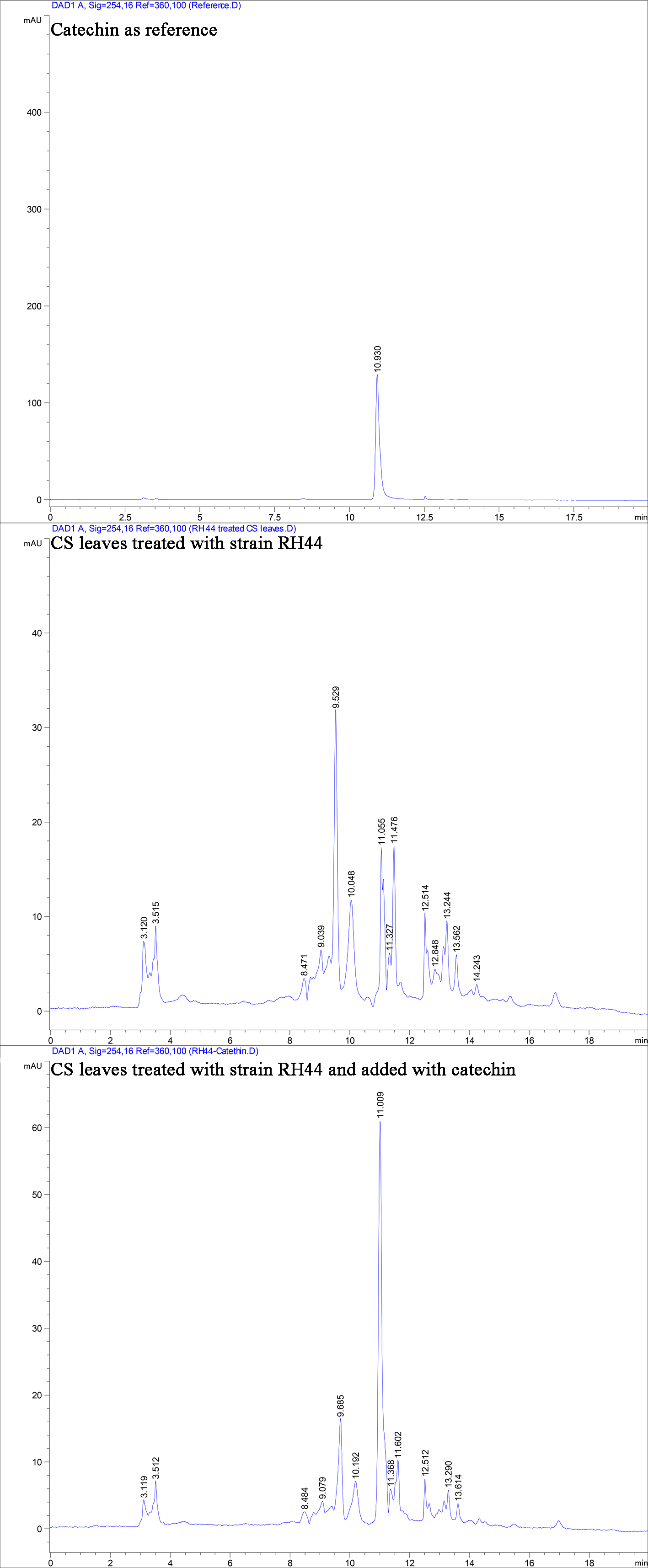

Supplement: S7 Fig — (TIF) [file pone.0238734.s011.tif]
